# Navigated transoral odontoidectomy to treat congenital basilar invagination after failed posterior reduction and fusion

**DOI:** 10.3171/2020.4.FocusVid.20158

**Published:** 2020-07-01

**Authors:** Wanru Duan, Dean Chou, Fengzeng Jian, Zan Chen

**Affiliations:** 1 Department of Neurosurgery, Xuanwu Hospital, Capital Medical University, Beijing, China; and; 2 Department of Neurological Surgery, University of California, San Francisco, California

**Keywords:** basilar invagination, atlantoaxial dislocation, transoral, odontoidectomy, revision surgery

## Abstract

Transoral odontoidectomy is a traditional technique to treat congenital basilar invagination (BI) associated with atlantoaxial dislocation (AAD). Although posterior surgery has been a trend to treat most cases, there are still cases that need to be treated through a transoral approach. In addition, intraoperative modern image-guided navigation systems help identify any remnants of the dens and decrease the risk of vertebral artery injury. For symptomatic cases with a history of previous posterior fusion and severe osteoporosis, transoral odontoidectomy is preferred over a posterior-only approach. Our video demonstrates the surgical technique for transoral revision odontoidectomy to treat congenital basilar invagination associated with atlantoaxial dislocation after previous posterior craniovertebral junction surgery.

The video can be found here: https://youtu.be/vzcAW8oLcZY

## Transcript

**0:20** This video demonstrates the surgical technique for transoral odontoidectomy to treat basilar invagination with atlantoaxial dislocation after a failed posterior occipital-cervical fusion to treat craniovertebral junction pathology.

**0:35** Clinical presentation. The patient is a 64-year-old woman who presented with progressive bilateral lower extremity weakness for 20 years and bilateral upper extremity weakness for 10 years. She suffered from stiffness of bilateral lower limbs and loss of dexterity of hands. Her symptoms worsened, and she developed dysphagia after posterior decompression and fusion 1 month ago.

Neurological examination revealed an unsteady gait, muscle strength grade 3–4 out of 5, and a left Babinski’s sign. Her JOA score was 10. The patient also had severe osteoporosis, a contraindication for revision posterior surgery (Duan et al., 2019). Thus, a transoral odontoidectomy was chosen to decompress the spinal cord (Wang and Yan, 2017; Mummaneni and Haid, 2005; Goel, 2005).

**1:31** Imaging. A midsagittal CT reconstruction showed that the atlantoaxial dislocation and the basilar invagination remain unreduced after the posterior surgery. The occipital plate had been implanted into the squamous part of occipital bone, which also had an assimilated C1.

The distance of the odontoid tip above Chamberlain’s line was 1.14 cm, the atlantodental interval was 3.06 mm, and the clivus-canal angle was 129.1°.

A sagittal T2-weighted MRI shows brainstem compression by the odontoid process. The surgical plan was an odontoidectomy through a transoral approach.

**2:18** Positioning. The patient was placed into the Mayfield skull clamp supine on the operating table, and standard endotracheal intubation was performed through the mouth. A transoral tongue retractor and a pharyngeal retractor were inserted to expose the oropharynx. The soft palate was suspended cranially using a slim silicone tube. The O-arm (Medtronic, Inc.) was used to obtain an intraoperative CT and register the neuronavigation (Brainlab).

**2:49** Exposure. A midline vertical incision was made using a blade dividing the mucosa. A navigated probe was used to locate both the base and the tip of the odontoid. Monopolar electrical cautery was used to divide the pharyngeal constrictor muscles and skeletonize the anterior C1 tubercle, the C1 arch, and the C2 vertebral body subperiosteally.

**3:21** Decompression. The center of the C1 arch and the C2 vertebral body were removed using a high-speed diamond burr. The navigational probe was used to identify the boundaries for drilling. Here, the navigation showed that further drilling was required to reach the cephalad-most tip of the odontoid. The cephalad-most portion of the odontoid was subsequently drilled until a thin rim of the odontoid was left. A microhook was then used to dissect the cortical shell away from the transverse ligament. A 2-mm Kerrison rongeur was subsequently used to remove the cortical shell. A Kerrison rongeur was used to remove the ligaments causing cord compression. A nerve hook was used to dissect any epidural tissue away from the spinal cord. The navigational probe was used to verify that the whole odontoid tip had been removed (Mummaneni and Haid, 2005; Tubbs et al., 2016).

**5:09** Closure. A piece of Gelfoam was placed over the dura. The muscle and mucosa were closed in layers with an absorbable suture.

**5:31** Postoperative imaging. A postoperative CT showed that the odontoid had been totally removed. An MRI showed good decompression of the brainstem. The patient noted improvement of her myelopathic symptoms on postoperative day 2.

## Acknowledgments

Funding from the following is acknowledged: Beijing Municipal Natural Science Foundation (Beijing Natural Science Foundation Grant 7172091), Beijing Municipal Administration of Hospitals (Beijing Municipal Administration of Hospitals Grant PX2017002), and Beijing Municipal Health Commission (Beijing Health Commission Independent Innovation Fund 2018-2-2014).
